# Increased IL-17 production correlates with immunosuppression involving myeloid-derived suppressor cells and nutritional impairment in patients with various gastrointestinal cancers

**DOI:** 10.3892/mco.2013.134

**Published:** 2013-05-27

**Authors:** TAKASHI YAZAWA, MASAHIKO SHIBATA, KENJI GONDA, TAKESHI MACHIDA, SATOSHI SUZUKI, AKIRA KENJO, IZUMI NAKAMURA, TAKAO TSUCHIYA, YOSHIHISA KOYAMA, KENICHI SAKURAI, TATSUO SHIMURA, RYOUICHI TOMITA, HITOSHI OHTO, MITSUKAZU GOTOH, SEIICHI TAKENOSHITA

**Affiliations:** 1 Departments of Organ Regulatory Surgery, Fukushima 960-1295;; 2 Tumor and Host Bioscience, Fukushima 960-1295;; 3 Blood Transfusion and Transplantation Immunology, Fukushima 960-1295;; 4 Immunology, Fukushima 960-1295;; 5 Regenerative Surgery, Fukushima 960-1295;; 6 Department of Surgery, Nihon University School of Medicine, Itabashi, Tokyo 173-8610;; 7 Department of Surgery, Nippon Dental University, Chiyoda, Tokyo 102-8158, Japan

**Keywords:** myeloid-derived suppressor cells, cachexia, gastrointestinal cancer, immune suppression, nutritional impairment

## Abstract

Although a causal relationship between inflammation and innate immunity of cancer is more widely accepted today, many of the precise cell mechanisms mediating this relationship have not been elucidated. Th17 cells, which produce the proinflammatory cytokine interleukin 17 (IL-17), have been recognized as one of the key factors in the regulation of inflammatory bowel disease and rheumatoid arthritis. This study demonstrated that, in patients with various types of gastrointestinal cancer, IL-17 production was correlated with myeloid-derived suppressor cell (MDSC) levels and with markers for nutritional impairment, immune suppression and chronic inflammation. IL-17 was significantly higher in patients with various types of gastrointestinal cancer compared to normal volunteers. In addition, IL-17 levels were significantly correlated with neutrophil counts and the neutrophil/lymphocyte ratio (NLR) and significantly inversely correlated with cell-mediated immune response indicators [lymphocyte phytohemagglutinin (PHA)-blastogenesis and IL-12 induction] and patient nutritional status (prealbumin levels). Circulating MDSC levels were significantly correlated with IL-17 production. These results suggest that, in human gastrointestinal cancers, chronic inflammation involving IL-17 may be an important mechanism contributing to disease progression through enhancement of immune suppression or cachexia. Controlling the activation of Th17 cells may prove to be a valuable strategy for the treatment of gastrointestinal cancer patients.

## Introduction

To the best of our knowledge, the functional relationship between inflammation and cancer has not been recently investigated. Karl Virchow hypothesized that cancer originates at sites of chronic inflammation (1). Although a causal relationship for inflammation and innate immunity of cancer is more widely accepted today, the precise cell mechanisms mediating this relationship have not been elucidated. Th17 cells were identified in 2005 (2–4) and in humans, the cytokines that direct Th17 cell lineage development likely include IL-6, IL-21, IL-23 and IL-1-β. In addition, TGF-β plays a potentially synergistic role through its ability to suppress Th1 cell lineage commitment (5,6). Although the IL-17 cytokine family includes six members, Th17 cells are considered to produce only the proinflammatory cytokines IL-17A and IL-17F, which are 55% identical. IL-17A and IL-17F combine to form a heterodimer (7). It was previously reported that IL-17 plays an important role in the pathogenesis of inflammatory bowel diseases (IBDs), including Crohn’s disease and ulcerative colitis (8,9).

Myeloid-derived suppressor cells (MDSCs) have been identified in the majority of patients and experimental mice with tumours and inflammation, based on their ability to suppress T-cell activation (10). In mice, MDSCs are uniformly characterised by expression of the cell surface molecules detected by antibodies to Gr1 and CD11b (11). Variations in the MDSC phenotype are consistent with the concept that MDSCs are a diverse family of cells that are in various intermediate stages of myeloid cell differentiation.

In humans, MDSCs are most commonly defined as CD14^−^CD11b^+^ cells or, more narrowly, as cells that express the common myeloid marker CD33 but not the markers of mature myeloid or lymphoid cells, or the MHC class II molecule HLA-DR (12). For the purposes of this study, MDSCs were defined as CD14^−^CD11b^+^CD33 cells.

Tumour development and growth occurs as a result of interactions between tumour and host immune/inflammatory cells and chronic inflammation plays an important role in cancer development and progression (13,14). Inflammatory parameters based on differential white cell counts, such as the neutrophil/lymphocyte ratio (NLR), may be simple and readily available biomarkers for tracking inflammation and cancer development. The results of the present study demonstrate the correlation of IL-17 production levels with MDSCs and other markers for nutritional status, immune suppression and chronic inflammation in patients with a variety of gastrointestinal cancers.

## Materials and methods

### Study subjects

Blood samples were collected from 60 patients with various types of gastrointestinal cancer, that were as follows: 7 esophageal (2 stage II, 2 stage III and 3 stage IV); 14 gastric (5 stage I, 3 stage II, 1 stage III and 5 stage IV); 20 colorectal (1 stage I, 7 stage II, 4 stage III and 8 stage IV); 5 hepatocellular (2 stage II and 3 stage III); 7 cholangiocellular (1 stage I, 2 stage III and 4 stage IV); and 7 pancreatic (2 stage II, 1 stage III and 4 stage IV) cancer patients. In addition, samples from 18 healthy volunteers of similar age and gender distributions were used as controls. The enrolled patients underwent surgery or chemotherapy for the treatment of histologically confirmed cancer in the departments of Organ Regulatory Surgery and Regenerative Surgery of Fukushima Medical University from January, 2011 to March, 2012. The patients were 41–85 years of age and newly diagnosed. Blood samples were collected prior to the intitiation of any treatment.

The study protocol was approved by the Ethics Committee of Fukushima Medical University (2010–2014) and written informed consent was obtained from the enrolled patients and normal donors.

### Blood samples

Peripheral blood mononuclear cells (PBMCs) were separated on Ficoll-Hypaque (Pharmacia-Biotech, Uppsala, Sweden) columns. The isolated PBMCs were washed twice with RPMI-1640 (Wako Pure Chemical Industries Ltd., Osaka, Japan) and maintained at −80°C in freezing medium (BLC-1; Juji-Field Co. Ltd., Tokyo, Japan) until used.

### Flow cytometry

**Figure 1:**
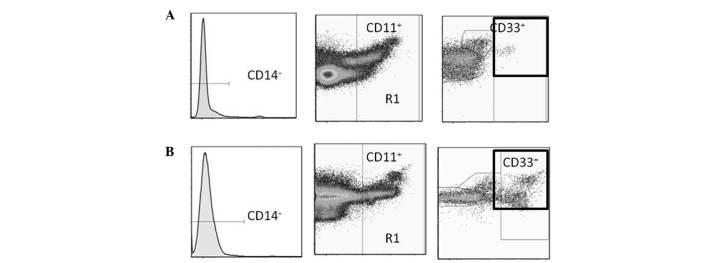
Immunophenotyping of myeloid-derived suppressor cells (MDSCs) by flow cytometry. Cells were labelled with fluorescent isothiocyanate (FITC), phycoerythrin (PE) and phycoerythrin cyanin 5.1 (PC5). Antibodies included those targeting FITC-conjugated CD14, PE-conjugated CD11b and PC5-conjugated CD33. (A) Healthy volunteer. (B) Gastric cancer patient.

Cells were labelled with fluorescent isothiocyanate (FITC), phycoerythrin (PE) and phycoerythrin cyanin 5.1 (PC5). Antibodies used included those directed against FITC-conjugated CD14 (Abcam, Cambridge, UK), PE-conjugated CD11b (Beckman Coulter, Inc., Marseille, France) and PC5-conjugated CD33 (Beckman Coulter), diluted in phosphate-buffered saline (PBS) to 10 and 50 *μ*g/ml. Cells were incubated with the antibodies for 20 min at 4°C and then washed with PBS. Data acquisition and analysis were performed using a FACSAria II flow cytometer (BD Biosciences, Mountain View, CA, USA) accompanied by Flow Jo software (TreeStar, Inc., Ashland, OR, USA). Typical expression patterns are shown in Fig. 1.

### Cytokine production by PBMCs

Samples (20 ml) of blood collected directly from heparinized collection tubes were subjected to Ficoll-density gradient centrifugation in order to isolate the PBMCs, 10^6^ of which were incubated in 1 ml of RPMI-1640 medium supplemented with 10% heat-inactivated fetal calf serum (Gibco BRL, Rockville, MD, USA) and 20 *μ*g/ml phytohemagglutinin (PHA) (Sigma, St. Louis, MO, USA) in 5% CO_2_ at 37°C for 24 h. Aliquots of these supernatants were then frozen and maintained at −80°C until use. Supernatant samples subsequently were thawed and used for measurement of IL-17 and IL-12 concentrations using ELISA test kits (R&D Systems, Minneapolis, MN, USA). Each sample was used only once after thawing.

### Lymphocyte proliferation assay

Lymphocyte proliferation assays were performed using PBMC suspended in RPMI-1640 (Wako Pure Chemical Industries, Ltd., Osaka, Japan) containing 10% fetal calf serum (Sigma). Following the addition of 10 *μ*g/ml PHA into PBMC culture wells kept at 37°C in a 5% CO_2_ atmosphere, PHA mitogenesis was observed for 80 h. ^3^H-thymidine (Japan Radioisotope Association, Tokyo, Japan) was added to the wells for the last 8 h of incubation. Cells were harvested and ^3^H-thymidine incorporation was determined using a liquid scintillation counter (PerkinElmer, Inc., Waltham, MA, USA) and expressed as counts per minute (cpm). The stimulation index (SI) was obtained by calculating total cpm/control cpm. The controls were defined as PBMCs that had not been subjected to PHA addition.

### Markers for nutritional status and chronic inflammation

To evaluate the nutritional status of the subjects, serum concentrations of albumin (determined by nephelometry) and prealbumin (determined using a turbidimetric immunoassay) were measured using standard protocols. Neutrophil and lymphocyte counts, as well as their ratios (NLR) in peripheral blood samples, were used as indicators of inflammation in this study.

### Statistical analysis

Differences between groups were determined by Student’s t-tests. Correlations between two variables were quantified by determining the Spearman’s rank correlation coefficients. P<0.05 was considered to indicate a statistically significant difference. Inadequate amounts of blood were obtained from some patients and in these cases, certain measurements were not possible.

## Results

### Factors affecting IL-7 production

**Figure 2:**
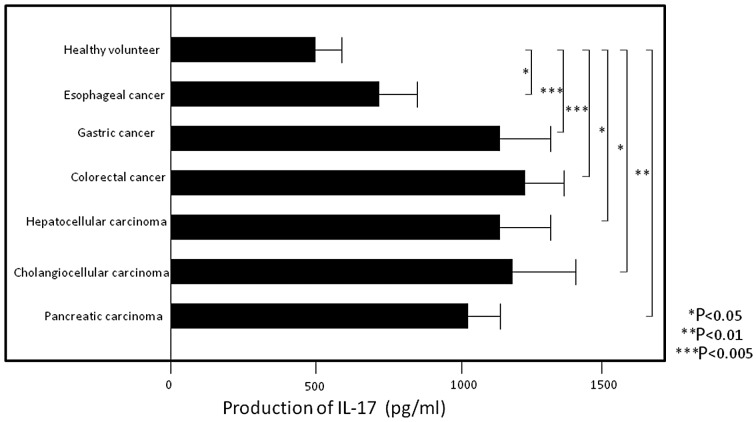
Production of interleukin 17 (IL-17) by peripheral blood mononuclear cells (PBMCs). In the investigated types of cancer, IL-17 production was significantly higher compared to that in healthy volunteers. Details regarding concentrations are provided in the text.

**Figure 3:**
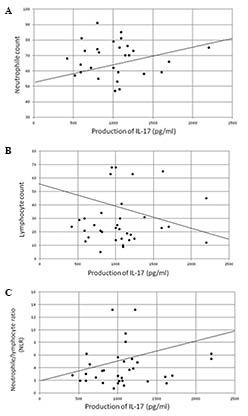
Correlation of interleukin 17 (IL-17) production with blood counts in various digestive cancers. IL-17 production was significantly correlated with (A) neutrophil count (P<0.005, r=0.436) and (C) the neutrophil/lymphocyte ratio (NLR) (P<0.005, r=0.535) and exhibited a significant inverse correlation with (B) lymphocyte count (P<0.01, r=−0.420).

**Figure 4:**
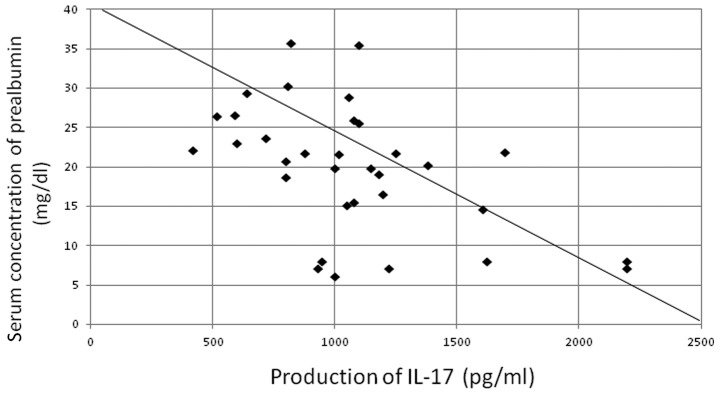
Correlation of interleukin 17 (IL-17) production with serum prealbumin concentration. IL-17 production was inversely correlated with serum prealbumin concentration, which is a marker for nutritional status (P<0.05, r=−0.387).

**Figure 5:**
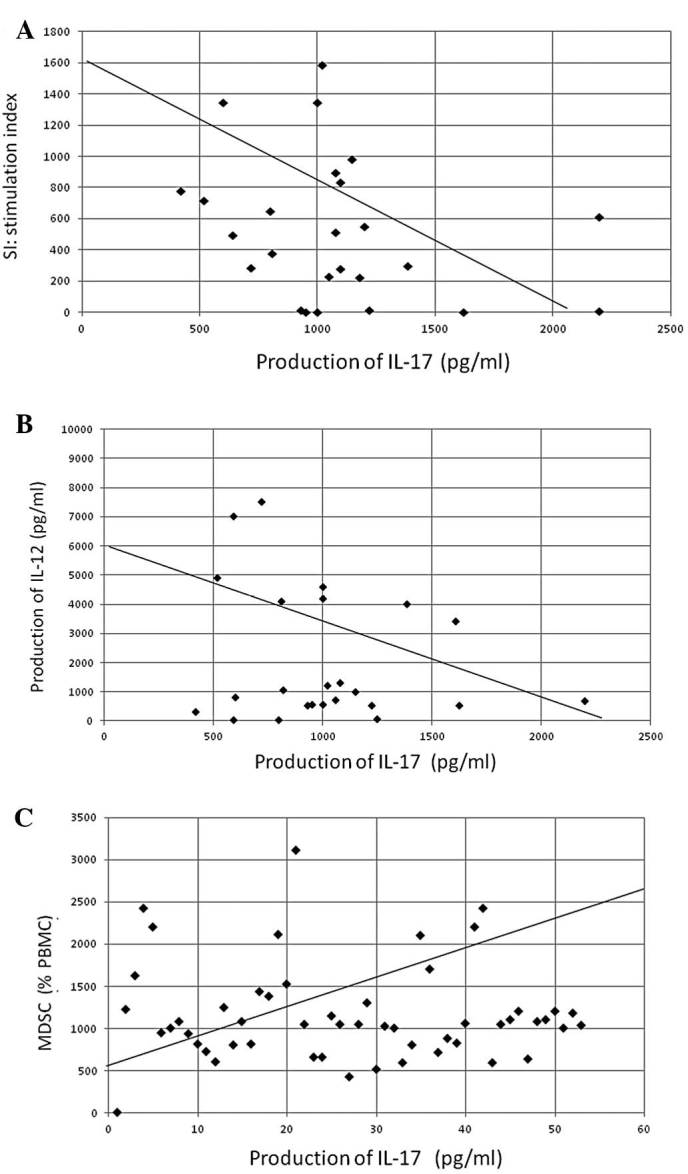
Correlation of interleukin 17 (IL-17) production with immunological parameters. IL-17 production was significantly inversely correlated with (A) stimulation indices of lymphocyte PHA-blastogenesis (P<0.05, r=−0.302) and (B) peripheral blood mononuclear cell (PBMC) IL-12 production (P<0.01, r=−0.411) and was significantly positively correlated with (C) circulating myeloid-derived suppressor cell (MDSC) levels (%PBMC, P<0.001, r=0.492).

PBMC IL-17 production levels were significantly higher in patients with esophageal (705.9±164.0, P<0.05), gastric (1167.1±135.6, P<0.005), colorectal (1231.4±145.5, P<0.005), hepatocellular (1133.6±212.6, P<0.05) cholangiocellular (1181.5±261.6, P<0.05) and pancreatic (1033.3±84.0, P<0.01) carcinoma compared to those in healthy volunteers (544.6±133.4) (Fig. 2) (all values are expressed as pg/ml). In addition, IL-17 production was significantly correlated with the neutrophil count (P<0.005, r=0.436) and NLR (P<0.005, r=0.535) and was significantly inversely correlated with the lymphocyte count (P<0.01, r=−0.420) (Fig. 3) and serum prealbumin concentration (P<0.05, r=−0.387) (Fig. 4). The production of IL-17 demonstrated a significant inverse correlation with SIs (found by assessing lymphocyte PHA-blastogenesis) (P<0.05, r=−0.302) and IL-12 production (P<0.01, r=−0.411), as well as a significant positive correlation with circulating levels of MDSCs (P<0.001, r=0.492) (Fig. 5).

## Discussion

To the best of our knowledge, this study is the first to describe an important role for IL-17 in the induction of immune suppression and nutritional impairment during systemic inflammation. IL-17 production was significantly higher in patients with various types of gastrointestinal cancer compared to that in normal volunteers. Production levels were significantly correlated with neutrophil counts and NLRs. By contrast, they were significantly inversely correlated with cell-mediated immune responses, including lymphocyte PHA-blastogenesis and Th1 induction, as reflected by IL-12 production and compromised patient nutritional status, as reflected by prealbumin levels. Circulating levels of MDSCs were also significantly correlated with IL-17 production levels.

Over the last decade, there has been an expansion of scientific knowledge regarding the pathogenesis of IBDs, including Crohn’s disease and ulcerative colitis. IBDs have been reported to arise due to a combination of genetic variations and alterations in intestinal microflora, which may subsequently promote an uncontrolled immune response and result in chronic intestinal inflammation (8,9). IL-17 is considered to stimulate various types of cells to produce proinflammatory mediators that amplify intestinal inflammation. It has also been reported that in humans, Th17 cells play an essential role in protective immunity against certain microorganisms (15). Th17 cells have a close developmental link with FOXP3^+^CD4^+^ regulatory T cells. Th17 cells have been reported to transiently express FOXP3 during their development (16). In the present study, the inflammation induced by IL-17 appeared to cause the production of MDSCs that may potentially inhibit maturation of dendritic cells, and cell-mediated immunity may be suppressed through Th2 dominant conditions driven by the depressed production of IL-12. Thus, Th17 cells may play important roles in the development of immune suppression in patients with malignant diseases.

The NLR was reported to be a marker of systemic inflammatory response and an independent predictor of clinical benefit, good prognosis and survival in patients receiving cancer chemotherapy (17). The likelihood of the association of IL-17 production with nutritional impairment is high, due to the role of IL-17 as a marker of systemic inflammation. It was previously reported that the key mechanisms leading to cancer cachexia, in which nutritional impairment is a major clinical issue, are mostly immune reactions caused by chronic inflammation and that treatment with a COX-2 inhibitor or a specific nutrient formula is effective (18,19).

Thus, in human cancers, chronic inflammation involving IL-17 is considered to be important in the development of disease-advancement indicators, such as immune suppression or cachexia. In order to suppress Th17, several candidates have been experimentally used. Since it was reported that Th17 cells are induced by TGF-β, IL-1, IL-6, IL-21 or IL-23, antibodies targeting IL-6 or IL-23 have been considered strong candidates for the development as treatments for autoimmune diseases, including IBD and rheumatoid arthritis (1,20). Further investigations are required to explore this possibility and gain more insight into this field of medicine.
